# Microbial physiological trait shifts link heavy metal remediation to enhanced soil carbon storage potential

**DOI:** 10.1093/ismejo/wrag250

**Published:** 2026-09-21

**Authors:** Rui Xue, Jian Li, Shilin Hu, Jiazhi Ding, Cheng Wang, Wenshun Ke, Chaoran Li, Philippe Corvini, Li Cui

**Affiliations:** State Key Laboratory of Regional and Urban Ecology, Institute of Urban Environment, Chinese Academy of Sciences, Xiamen, Fujian 361021, China; College of Resources and Environment, University of Chinese Academy of Sciences, 19A Yuquan Road, Beijing 100049, China; Ministry of Education Key Laboratory of Ecology and Resource Use of the Mongolian Plateau & Inner Mongolia Key Laboratory of Grassland Ecology, School of Ecology and Environment, Inner Mongolia University, Hohhot, Inner Mongolia 010021, China; State Key Laboratory of Regional and Urban Ecology, Institute of Urban Environment, Chinese Academy of Sciences, Xiamen, Fujian 361021, China; Department of Environmental Science and Engineering, University of Science and Technology of China, Hefei, Anhui 230026, China; State Key Laboratory of Regional and Urban Ecology, Institute of Urban Environment, Chinese Academy of Sciences, Xiamen, Fujian 361021, China; College of Resources and Environment, University of Chinese Academy of Sciences, 19A Yuquan Road, Beijing 100049, China; Environmental Microbiomics Research Center, School of Environmental Science and Engineering, Southern Marine Science and Engineering Guangdong Laboratory (Zhuhai), Sun Yat-sen University, Guangzhou, Guangdong 510006, China; College of Environment and Ecology, Hunan Agricultural University, Changsha, Hunan 410128, China; State Key Laboratory of Crop Stress Adaptation and Improvement, Henan University, Kaifeng, Henan 475004, China; Institute for Chemistry and Bioanalytics, FHNW University of Applied Sciences and Arts Northwestern Switzerland, 4132 Muttenz, Switzerland; State Key Laboratory of Regional and Urban Ecology, Institute of Urban Environment, Chinese Academy of Sciences, Xiamen, Fujian 361021, China

**Keywords:** heavy metal, soil remediation, soil carbon cycle, microbial ecology, single-cell Raman spectroscopy

## Abstract

Widespread and chronic heavy metal pollution resulting from industrial activities has compromised the sustainability of soil ecosystems. Increasing and stabilizing soil carbon storage is central to soil development, but how remediation reshapes soil carbon cycling processes during the mitigation of heavy-metal contamination remains unclear. Here, we tracked genome-scale microbial metabolism, community turnover, and phenotype-level physiological responses during a 120-day remediation of heavy metal-contaminated soils with several decades of pollution histories and resolved their dynamic interplay with microbial carbon use efficiency (CUE) and CO_2_ emissions. We found that heavy-metal stress accelerated microbial respiratory carbon loss from soils, with contaminated soils exhibiting significantly higher cumulative CO₂ emissions than both nearby uncontaminated and remediated soils. Metagenomic profiles were enriched in oxidative-stress defense and metal-detoxification functions, consistent with elevated maintenance costs that may contribute to enhanced respiration. In contrast, remediation significantly reduced soil CO₂ emissions while increasing microbial growth rate and CUE, indicating a shift toward greater soil carbon storage potential. Raman-based *in situ* monitoring further showed that biomolecules associated with microbial growth, including phospholipids, nucleic acids, and proteins, increased progressively throughout the remediation process. Structural equation modelling further revealed that microbial physiological traits, particularly metabolic activity and intracellular biomolecular content, exerted stronger direct effects on CUE than community traits, including community stability and life-history strategy. These results identify microbial physiological traits as a key link between environmental stress and soil carbon cycling. Together, these findings suggest that remediating heavy metal-contaminated soils may represent an underappreciated pathway for enhancing terrestrial carbon sequestration.

##  Introduction

Soil heavy metal contamination has long been recognized as a persistent and severe global environmental problem [1, 2]. It severely disrupts microbial metabolism and biogeochemical cycling, thereby diminishing soil productivity and compromising the long-term capacity of soils to sustain essential ecosystem functions [3, 4]. Soil organic carbon (SOC) is a core component underpinning soil functions, playing a pivotal role in stabilizing soil structure, regulating nutrient cycling, and sustaining microbial activity and diversity [5]. The fate of SOC is closely intertwined with microbial metabolic processes. Microbial carbon use efficiency (CUE), the fraction of assimilated carbon allocated to biomass production rather than respiration, is a key parameter for predicting global soil carbon stocks [6, 7]. Currently, the objectives of soil remediation have shifted from a sole focus on efficient heavy metal immobilization or removal toward strategies that promote robust and sustainable remediation, emphasizing both environmental safety and ecosystem function sustainability [8]. Therefore, gaining a deeper understanding of the changes in microbial CUE and CO_2_ emissions during the remediation of heavy metal-contaminated soils is essential for predicting soil development dynamics and guiding effective remediation strategies. This will ultimately facilitate the restoration and sustainable reuse of abandoned historically contaminated lands.

Heavy metals can disrupt microbial metabolism through various biochemical mechanisms. One key mechanism involves the displacement of essential metal cofactors in specific enzymes, ultimately causing microbial metabolic dysfunction or even cell death [9]. In environments with long-term heavy metal contamination, microbial communities have evolved a range of resistance strategies to mitigate metal toxicity, including active efflux, intracellular sequestration, and chemical transformation of metal species [10]. The activation of these resistance mechanisms may reallocate cellular resources and energy from biomass production toward stress tolerance and maintenance. Such investment can increase energetic demands and maintenance-associated respiratory costs, thereby reducing the fraction of assimilated carbon allocated to growth and potentially lowering microbial CUE, ultimately leading to adverse consequences for soil carbon storage. Although soil remediation is a key strategy for alleviating heavy metal contamination, historical contamination may impose persistent constraints on microbial carbon metabolism even after the stressor is mitigated [11–13]. This potential mismatch between the timescales of contaminant mitigation and the recovery of microbial carbon metabolism is particularly relevant to the rapid redevelopment and long-term sustainable use of contaminated sites. Such sites are increasingly converted to urban green spaces and other regenerated land uses [8], which can constrain remediation to relatively short operational time frames. Whether short-term remediation can simultaneously reduce heavy metal stress and counteract the persistent effects of historical contamination on microbial carbon metabolism remains unclear. This knowledge gap hinders our ability to predict SOC accumulation potential and soil function recovery in remediated soils.

Current theory increasingly conceptualizes microbial CUE as an emergent property arising from underlying metabolic and physiological processes, rather than as a single physiological parameter [14]. At the community level, variation in microbial CUE therefore reflects interacting effects of soil physicochemical conditions, microbial community composition, and physiological states [15–17]. However, attempts to identify the microbial traits underlying variation in CUE have often relied on sequence-based characterization of microbial community composition and functional potential [15, 16]. Because the transition from genotype to effective phenotype is mediated by transcriptional, translational, and post-translational regulation, gene abundance alone may provide an incomplete proxy for realized physiological activity and biogeochemical process rates [18]. This limits our ability to identify the effective microbial traits that govern soil microbial CUE. Directly quantifying these effective traits in situ is therefore essential but remains a major methodological challenge. Raman spectroscopy enables non-destructive characterization of whole-cell biomolecular fingerprints at single-cell resolution. When combined with D₂O labeling, it detects deuterium incorporation into newly synthesized cellular biomolecules, providing a quantitative proxy for single-cell metabolic activity and enabling estimation of the fraction of active cells [19]. This approach enables direct characterization of cell-resolved microbial physiological traits that are obscured by community-averaged measurements, thereby broadening our understanding of microbial controls on soil carbon cycling.

Here, we investigated the dynamics of microbial CUE and CO_2_ emissions over a 120-day soil remediation process. We expected that alleviation of heavy-metal stress during remediation would shift microbial carbon allocation between respiration and biomass production, and that these community-level changes would be accompanied by shifts in active bacterial community composition and cell-resolved physiology. To evaluate these expectations, we integrated metagenomics, fluorescent D-amino acid (FDAA) labeling coupled with fluorescence-activated cell sorting and sequencing, and single-cell Raman-D₂O analysis to (i) quantify temporal changes in CO_2_ emissions, microbial growth, and CUE throughout remediation; (ii) elucidate the genomic mechanisms underlying remediation-induced shifts in microbial CO_2_ emissions; and (iii) characterize the temporal changes in active bacterial community composition and physiological traits during remediation and assess their relative importance in explaining variation in microbial CUE. Together, this integrated framework provides new insights into how remediation reshapes microbial carbon allocation and physiology during recovery from long-term Pb, Cd, and As co-contamination.

## Materials and methods

### Site description and experimental design

The sampling site was located in an abandoned zinc smelting area in Zhuzhou City, Hunan Province, China, where smelting activities had operated for 60 years until production ceased in 2018. The region is characterized by a subtropical monsoonal climate, with a mean annual temperature of 17°C. We collected soils representing three levels of contamination (L, M, and H) from the study area, ranked according to total heavy metal concentrations as L < M < H. In addition, three uncontaminated soils (N1, N2, and N3) were collected from nearby surrounding areas (Table S1). The soils were air-dried and passed through a 2-mm sieve to remove debris. A ferrous sulfide nanomaterial used as the soil remediation agent was obtained from Henan University. A total of 1000 g of each soil (L, M, and H) was placed in a plastic pot. The experiment comprised two consecutive phases, with remediation conducted from Day 0 to Day 60 and ryegrass cultivation from Day 60 to Day 120 to simulate the sequential process of soil ecological restoration. During the first phase (Days 0-60), soils assigned to the remediation treatment were amended with 3% (w/w) ferrous sulfide nanomaterial, which had been sterilized in 70% (v/v) ethanol for 30 min. In parallel, FeS-unamended control soils (CK) of each soil type were maintained under the same conditions. All soils were adjusted to 70% of their maximum water-holding capacity with deionized water and incubated at 25°C for 60 days. During the second phase (Days 60-120), surface-sterilized ryegrass seeds (30% (v/v) H₂O₂ for 30 min) were evenly sown into both the FeS-amended and FeS-unamended soils. After germination, the seedlings were grown for a further 60 days under the same natural-light and ambient-humidity conditions. Deionized water was applied regularly to maintain all soils at 70% of their maximum water-holding capacity throughout the 120-day experiment. To assess whether FeS itself directly affected microbial carbon metabolism, an additional 30-day control experiment was conducted using three uncontaminated soils. For each soil, FeS-amended and unamended treatments were established using the same FeS dosage and incubation conditions as described above. At the end of the incubation, cumulative CO₂ release, microbial respiration rate, microbial growth rate, and CUE were determined using the same methods as in the main experiment. DNA sequencing was not performed on these control soils because microbial biomass, respiration, growth, and metabolic activity remained stable throughout the incubation, indicating limited temporal variation in microbial activity and functional shifts.

Soil samples were collected at three time points: Day 0 (polluted state), Day 60 (end of chemical remediation), and Day 120 (end of ecological remediation), representing a typical ecological restoration process of contaminated soil following anthropogenic intervention. Each sample was divided into five aliquots for: (i) Physicochemical properties analysis, (ii) ^13^C-glucose labeling, (iii) Raman phenotype analysis, (iv) FDAA labeling, and (v) DNA extraction and sequencing.

### Soil physicochemical analysis

Soil pH was determined in a soil-water suspension (1:2.5, w/v) using a digital pH meter. SOC was measured by potassium dichromate oxidation with external heating. Dissolved organic carbon (DOC) was extracted from fresh soil with 0.05 M K₂SO₄ and determined using a TOC analyzer (TOC-LCPH). Ammonium (NH₄^+^) and nitrate (NO₃^−^) were extracted with 2 M KCl and analyzed with an AA3 continuous-flow analyzer. Microbial biomass carbon (MBC) was determined by the chloroform fumigation-extraction method using 0.05 M K₂SO₄ as the extractant, and was calculated as the difference in extractable C between fumigated and non-fumigated soils divided by k_EC_ = 0.45 [20]. Basal soil respiration was determined from headspace CO₂ accumulation in 60-ml airtight vials. Available phosphorus was extracted with 0.5 M NaHCO₃ (pH = 8.5) and measured by ICP-OES.

Bioavailable As was extracted with 0.5 M NaHCO₃, whereas bioavailable Cd and Pb were extracted using a DTPA-CaCl₂-TEA solution (0.005 M DTPA, 0.01 M CaCl₂, 0.1 M TEA, pH 7.3); all extractions were performed at a 1:5 (w/v) ratio with 2 h shaking at 25°C, and metal concentrations were determined by ICP-OES (Agilent, USA).

### Analysis of soil CO_2_ efflux and microbial carbon use efficiency

To quantify the effects of remediation on biogenic soil CO₂ emissions, 20 g of each contaminated soil and its corresponding remediation-amended soil were placed in 1-L sealed plastic bottles, incubated in the dark for 60 days, and then analyzed for cumulative CO₂ emissions. Soil microbial CUE was quantified using a substrate-specific ^13^C-glucose tracing assay. Glucose was added at 0.05 mg C g^−1^ soil, with a final ^13^C enrichment of 25 atom% achieved by mixing 99 atom% uniformly labeled ^13^C-glucose with unlabeled glucose. For each treatment, three tracer-amended replicates (4 g soil per replicate) and three tracer-free controls were prepared. After amendment, soils were gently homogenized, transferred to 60-ml headspace vials, and the headspace was flushed with CO₂-free air to standardize initial conditions. The vials were then incubated at 25°C for 48 h. Detailed experimental procedures, isotopic partitioning, and CUE calculations are provided in Text S1.

### Phenotypic characterization of soil bacteria via Raman-D₂O labeling and cell recovery

Fresh soil was air dried at room temperature for 24 h to eliminate excess moisture. Then we added 200 μL D_2_O (99 atom% D, Sigma–Aldrich) containing 0.125 mg glucose to 1 g of soil, followed by incubation at 25°C for 48 h. Non-labelled H₂O controls received the same glucose amendment and were incubated for 48 h under the same conditions. After incubation, soil microorganisms were isolated by Nycodenz density-gradient separation. Soils were suspended in phosphate-buffered saline containing 0.02% Tween 20 at a 1:5 (w/v) soil-to-solution ratio and vortexed for 30 min. Detached bacterial cells were separated from soil particles by Nycodenz density-gradient centrifugation [21]. The resulting slurry was layered onto 5 ml of 0.8 g ml^−1^ Nycodenz solution (≥ 98%, Aladdin), and centrifuged at 10 000 × *g* for 60 min. Following centrifugation, bacterial cells accumulated in the interfacial band between the aqueous soil suspension and the Nycodenz layer, and the interfacial layer was recovered and washed three times with deionized water.

### Single-cell Raman spectra acquisition and data analysis

Soil bacteria were mounted on an aluminum-coated slide (HOOKE Instruments Ltd., China) and air dried at room temperature prior to Raman analysis. Single-cell Raman spectra were obtained using a LabRAM Aramis confocal Raman microscope (HORIBA Jonin-Yvon, Japan) configured with a 532-nm Nd:YAG excitation source (Laser Quantum) and a 300 grooves/mm diffraction grating. Raman spectral collection was performed with a 100× objective lens (Olympus, Japan), using an integration time of 15 s per individual cell across 400–3200 cm^−1^.

For each soil sample, 270 spectra were collected. Raw data were baseline-corrected and normalized using LabSpec 6 (Horiba Scientific, France). The C-D (2040-2300 cm^−1^) and C-H (2800-3100 cm^−1^) regions were integrated to calculate CD/(CD + CH) ratios as a proxy for bacterial metabolic activity [22], with cells classified as metabolically active when their ratios exceeded the mean + 3 SD of the non-labelled controls. For Raman fingerprinting analysis, relative peak intensity, used as a semiquantitative proxy for intracellular biomolecular content in bacterial cells, was calculated as the sum of the maximum signal intensity and the intensities of the adjacent channels, following previously described methods [23].

### Fluorescent D-amino acid labeling and flow cytometry

FDAAs have emerged as a powerful tool for identifying active microbes by labeling the peptidoglycan of growing bacterial cells, thereby enabling precise tracking of metabolically active populations, and have been used to investigate active bacterial community composition in gut microbiomes [24]. In this study, we employed fluorescent D-amino acids (FDAA) to selectively label active bacteria in soil. Specifically, each sample (1 g soil) was incubated with 2 ml of 200 μM 5′-carboxyfluorescein (FAM) labeled amino-D-alanine (FADA) (Chinese Peptide Company, Hangzhou, China) in sterile water at 25°C in the dark for 48 h under gentle shaking to minimize oxygen limitation and diffusional constraints on FADA availability. At the end of the incubation period, soil microorganisms were extracted using the Nycodenz density-gradient separation method.

Prior to sorting, the extracted cell suspensions were filtered through a 40 μm cell strainer and analyzed using an S3e Cell Sorter (Bio-Rad) equipped with a 488 nm laser for FDAA excitation. For each sample, 500 000 bacterial events were acquired at an event rate of approximately 1000 events s^−1^ and analyzed using FlowJo. FDAA-labeled active cells were gated based on fluorescence thresholds established using no-FDAA controls (Figs S1–S2).

### DNA extraction, sequencing, and analysis

Total DNA was extracted from 0.5 g of soil using the FastDNA Spin Kit for Soil (MP Biomedicals) following the manufacturer’s protocols. Active bacterial DNA was extracted from sorted cells using a bacterial DNA extraction kit (CWBIO, China) according to the manufacturer’s protocols. DNA quality and concentration were assessed by 1.0% agarose gel electrophoresis and NanoDrop 2000 spectrophotometry. Total DNA was used to prepare metagenomic libraries, which were sequenced on a DNBSEQ-T7 platform (Wefind Biotechnology, Wuhan). The assembly and annotation of metagenomes are described in detail in Text S2. Bacterial community composition was assessed by 16S rRNA gene amplicon sequencing of DNA extracted from the sorted cells using the primer pair 515F (5′-GTGCCAGCMGCCGCGGTAA-3′) and 806R (5′-GGACTACHVGGGTWTCTAAT-3′). The PCR mixture contained Fast Pfu buffer, dNTPs, primer, Fast Pfu polymerase, template DNA, and ddH_2_O. PCR was performed at 95°C for 3 min, followed by 29 cycles of 95°C for 30 s, 53°C for 30 s, and 72°C for 45 s, ending with a final extension at 72°C for 10 min. The PCR products were sequenced using the Illumina MiSeq System (Illumina, San Diego, CA, USA), using 2 × 300-bp paired-end reads.

After demultiplexing, the resulting sequences were quality filtered with fastp (0.19.6) and merged with FLASH (v1.2.11). Then the high-quality sequencing data were processed in QIIME 2, where denoising was performed using the DADA2 plugin. Taxonomic classification was conducted against the SILVA v138.2 database.

### Calculation of active bacterial life history trait and community stability

Active bacterial life-history strategy was represented by the community-weighted mean rRNA operon (*rrn*) copy number. Briefly, the *rrn* copy number assigned to each ASV was inferred from its closest related taxon with available *rrn* information in the rrnDB database (v5.7), and sample-level mean *rrn* copy number was calculated by weighting each ASV-specific *rrn* copy number by its relative abundance in that sample. Active bacterial community stability was estimated by the ratio of negative to positive cohesion (|negative cohesion|/positive cohesion). Cohesion was calculated using a workflow based on previously described methods [25, 26].

### Statistical analysis

Differences among groups were assessed by one-way analysis of variance (ANOVA), followed by Tukey’s honestly significant difference (HSD) test for multiple comparisons, with *P* < .05 considered statistically significant. For metagenomic functional profiles, differential features were first tested by one-way ANOVA, and the resulting *P* values were corrected for multiple testing using the Benjamini–Hochberg false discovery rate procedure, with BH-adjusted *P* < .05 considered significant. Differences in bacterial community composition were evaluated by principal coordinates analysis (PCoA) and Adonis analysis. Spearman correlations were calculated in R v4.4.1 using the psych package, whereas PCoA and Adonis analyses were performed using the vegan package. We used partial least squares regression (PLSR) to evaluate the relative importance of Raman fingerprints associated with the microbial CUE, and all measured Raman fingerprint variables were included as predictors. PLSR was conducted in JMP Pro (version 16.1.0) using the NIPALS algorithm. Predictor variables with a VIP score greater than 1 were retained in the final models. To test the hypothesized relationships between microbial CUE and its drivers, we used SEM implemented through the “piecewiseSEM” R package. Given the strong collinearity among heavy metal variables and among intracellular bacterial biomolecular variables within each group, principal component analysis (PCA) was performed before SEM construction. The first principal component (PC1) was retained to generate a heavy metal composite index and a biomolecular composite index, explaining 91.0% and 96.2% of the total variance, respectively, and these PC1 scores were then used as composite variables in the subsequent SEM analysis. Fisher’s C statistic was used to assess overall model fit; *P* value >.05 was interpreted as no evidence of lack of fit. Other plots were generated using the ggplot2 package on R version 4.4.1.

## Results

### Long-term heavy metal contamination is associated with higher cumulative CO₂ emissions and lower soil organic carbon

We first examined how cumulative CO_2_ emissions and SOC varied across three heavy metal**-**contaminated soils and three uncontaminated reference soils collected around a legacy lead**-**zinc smelter. Cumulative CO₂ emissions were consistently higher in contaminated than in uncontaminated soils, with an average increase of 79.4%. The highest cumulative CO₂ emissions were observed in the heavily contaminated soil (167.3 μg CO₂-C g^−1^ soil; Fig. 1A). In contrast, SOC contents were significantly lower in contaminated soils than in the adjacent uncontaminated reference soils (Fig. 1B). Across all soils, cumulative CO_2_ emissions were negatively associated with NH₄^+^ and available phosphorus, positively associated with bioavailable heavy metals (BA-HMs), but not significantly associated with NO₃^−^ and pH (Fig. 1C). SOC showed the opposite pattern, being positively associated with NH₄^+^ and available phosphorus, negatively associated with BA-HMs, but not significantly associated with NO₃^−^ and pH (Fig. 1D). Together, these results show that long-term heavy metal contamination was associated with elevated cumulative CO_2_ emissions and reduced SOC contents. We hypothesized that the elevated cumulative CO_2_ emissions in heavy metal-contaminated soils arose from the energetic costs of metal detoxification, and that remediation would decrease soil CO₂ emissions by reducing metal bioavailability and thereby relieving microbial maintenance demand. To test this hypothesis, we established a soil remediation experiment and tracked key carbon-cycling processes over time.

**Figure 1:**
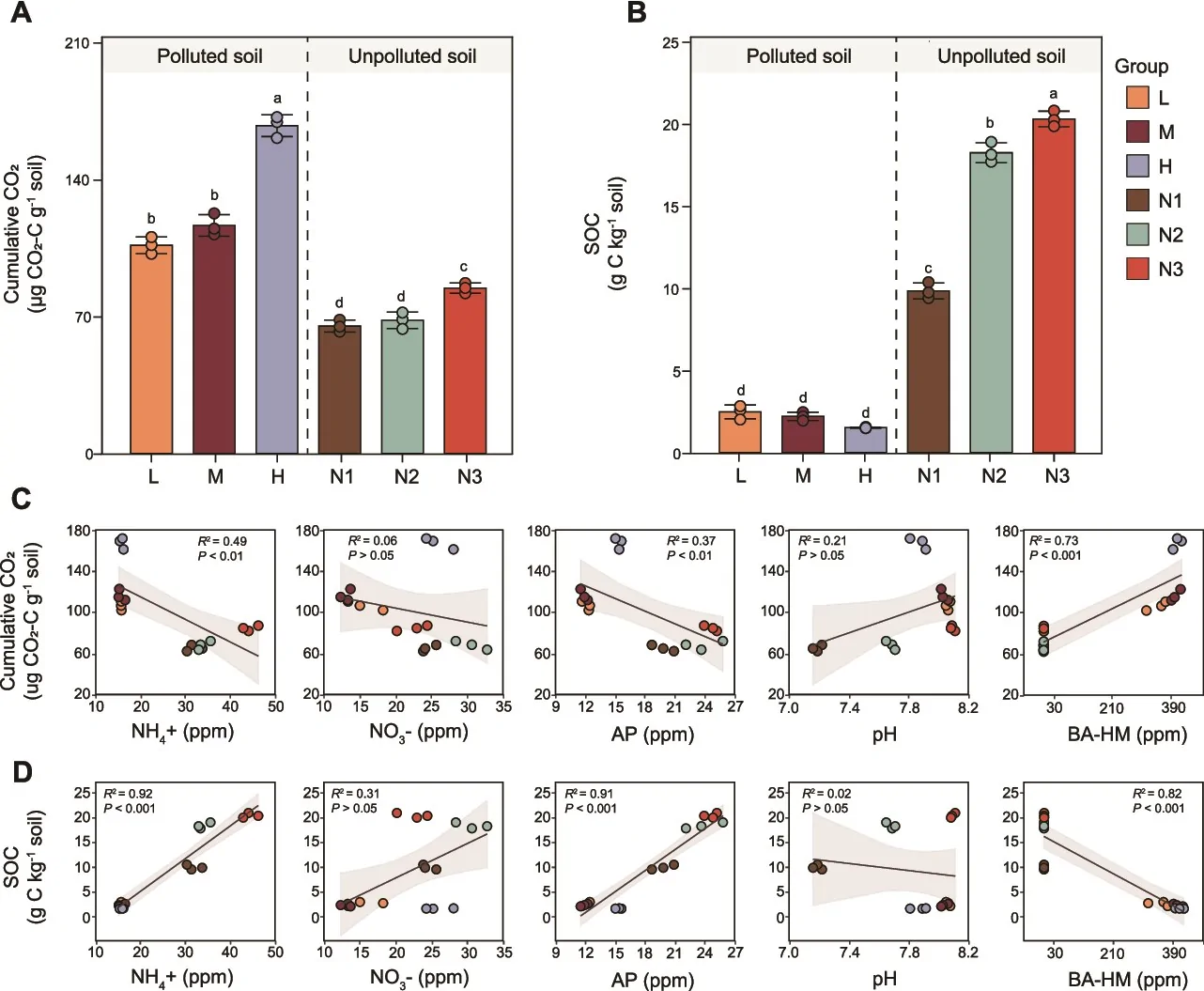
Cumulative CO₂ emissions and SOC across polluted and unpolluted soils. (A) Cumulative CO₂ emissions during the 30-day incubation and (B) SOC content in polluted soils (L, M, and H) and adjacent unpolluted soils (N1, N2, and N3). Bars represent the mean ± SD (*n* = 3). Different letters above the bars indicate significant differences among treatments, as determined by one-way ANOVA followed by Tukey’s HSD test (*P* < .05). (C) Spearman correlation analysis between cumulative CO₂ emissions and soil NH₄^+^, NO₃^−^, available phosphorus (AP), pH, and BA-HMs. (D) Spearman correlation analysis between SOC content and soil properties (NH_4_^+^, NO_3_^−^, available phosphorus (AP), pH, and BA-HMs). The black line shows the linear regression fit with 95% confidence intervals (gray shading). ppm values indicate concentrations on a dry-soil mass basis (mg kg^−1^ dry soil).

### Remediation simultaneously reduced bioavailable heavy metals and cumulative CO₂ emissions

We used a short-term microcosm experiment to simulate two consecutive stages of soil ecological restoration, with remediation conducted during the first 60 days, followed by ryegrass cultivation from Day 60 to Day 120 (Fig. 2A). Across all three contamination levels, remediation significantly reduced BA-HM concentrations (Fig. S3) and lowered cumulative soil CO₂ emissions by Day 60 (Fig. 2B). Relative to the corresponding non-treated soils, cumulative CO₂ emissions decreased by 27.0%, 24.2%, and 51.2% in low-, moderate-, and high-contamination soils, respectively. We further assessed ryegrass biomass in contaminated and remediated soils. Aboveground biomass was significantly higher in remediated soils than in non-treated contaminated soils, with ryegrass in non-treated soils producing only ~5.3% of the biomass measured in remediated soils (Fig. S4).

**Figure 2:**
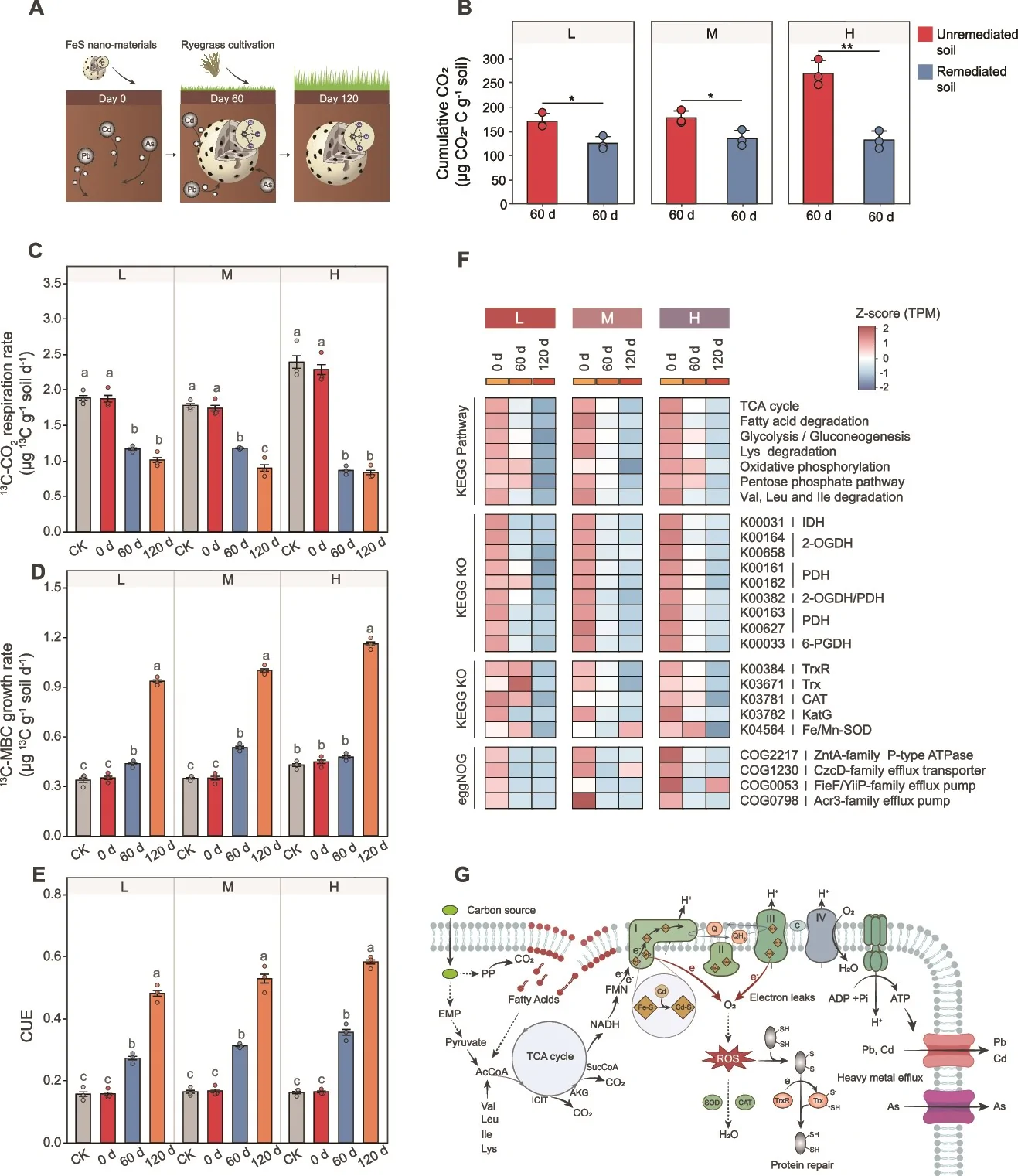
Effects of remediation on the microbial carbon metabolism and metagenomic features associated with increased respiration in L,M, and H soils. (A) Schematic illustration of the soil remediation process. Day 0 represents the non-treated contaminated soil. By Day 60, contaminated soils had undergone 60 days of nano-FeS remediation, after which ryegrass was planted and grown until Day 120. (B) Effects of remediation on cumulative CO_2_ emissions from soil. Significance was assessed using Student’s *t-*test (^*^P < .05, ^**^P < .01). (C–E) Temporal changes in microbial respiration rate, microbial growth rate, and microbial CUE during the remediation process. CK represents the contaminated control soil without remediation material amendment, which was planted with ryegrass from Day 60 to Day 120 and sampled at Day 120. Bars represent the mean ± SD (*n* = 3). Different letters indicate significant differences among treatments, as determined by one-way ANOVA followed by Tukey’s HSD test (*P* < .05). (F) Heat map showing metagenome-inferred relative abundance of pathways involved in microbial central carbon metabolism and associated processes directly linked tomicrobial respiration and oxidative stress, based on KEGG annotation, together with heavy metal efflux pathways based on eggNOG annotation. Values are shown as Z-scores calculated from TPM-normalized abundances (TPM, transcripts per million, used here as a gene-length- and sequencing-depth-normalized abundance metric). (G) Schematic overview of pathways related to microbial respiration, oxidative stress, energy metabolism, and heavy metal efflux. MBC, microbial biomass carbon; TCA cycle, tricarboxylic acid cycle; EMP, Embden-Meyerhof-Parnas pathway; PP, pentose phosphate pathway; Val, valine; Leu, leucine; Ile, isoleucine; Lys, lysine; AcCoA, acetyl-CoA; SucCoA, succinyl-CoA; ICIT, isocitrate; AKG, alpha-ketoglutarate; FMN, flavin mononucleotide; ROS, reactive oxygen species. The KEGG KOs shown here represent key enzymatic components of central carbon metabolism and oxidative stress responses. K00031, IDH (isocitrate to 2-oxoglutarate and CO₂); K00164 and K00658, 2-oxoglutarate dehydrogenase complex components, with K00382 encoding the shared E3 dihydrolipoyl dehydrogenase component involved in NAD^+^-dependent redox recycling; K00161, K00162, K00163, and K00627, pyruvate dehydrogenase complex components, which catalyze pyruvate conversion to acetyl-CoA and CO₂; and K00033, 6-phosphogluconate dehydrogenase (6-PGDH; 6-phosphogluconate to ribulose-5-phosphate and CO₂). Oxidative stress-related KOs include K00384, thioredoxin reductase; K03671, thioredoxin; K03781, catalase; K03782, catalase-peroxidase; and K04564, Fe/Mn-superoxide dismutase.

Remediation reduced CO₂ release without apparent impairment of microbial viability. Our results showed that microbial respiration declined after 60 days of remediation, whereas microbial growth rate and CUE increased across all contamination levels (Fig. 2C–E). In low-contamination soils, microbial respiration was 37.8% lower than at Day 0, whereas microbial growth and CUE increased by 24.9% and 73.0%, respectively. Similar patterns were observed in the moderate- and high-contamination soils. An independent control experiment further showed that nano-FeS addition had no significant effects on cumulative CO₂ emissions, microbial respiration, growth rate, and CUE across three uncontaminated soils (Fig. S5), suggesting minimal direct effects of nano-FeS on microbial carbon metabolism. From Day 60 to Day 120, ryegrass cultivation was associated with increased microbial growth and CUE, whereas microbial respiration rates remained relatively low (Fig. 2C–E). In addition, MBC showed a sustained increase throughout remediation, whereas the microbial metabolic quotient decreased consistently across all samples, indicating a reduction in microbial metabolic burden as remediation progressed (Fig. S6). These results suggest that remediation alleviated soil carbon loss while promoting more efficient microbial growth.

To investigate the microbial functional potential underlying the decrease in soil CO₂ release during remediation, we used a gene-centric metagenomic approach to profile genes involved in central carbon metabolism, energy production, oxidative stress defense, and metal detoxification, because these functions encompass key pathways involved in respiratory CO₂ production and ATP-dependent microbial heavy-metal detoxification (Fig. 2F). Across all three contamination levels, soils at Day 0 showed higher aggregated relative abundances of genes assigned to oxidative phosphorylation and the tricarboxylic acid (TCA) cycle, and these pathway-level abundances declined progressively during remediation (Table S2). Consistent with the pathway-level patterns, the relative abundances of genes encoding key oxidative decarboxylation enzymes, including isocitrate dehydrogenase (IDH), 2-oxoglutarate dehydrogenase (2-OGDH), pyruvate dehydrogenase (PDH), and 6-phosphogluconate dehydrogenase (6-PGDH), followed a similar decreasing trend during remediation (Fig. 2F, Table S3). These genes encode enzymes catalyzing central oxidative decarboxylation reactions that release CO₂, and their temporal trends in abundance were strongly correlated with the measured soil respiration rates during remediation (Fig. S7).

Genes involved in fatty-acid degradation, glycolysis/gluconeogenesis, and the degradation of lysine and branched-chain amino acids were also relatively enriched at Day 0 and decreased during remediation (Fig. 2F, Table S2), suggesting greater genomic potential for substrate catabolism and central carbon metabolism that can supply intermediates to the TCA cycle under heavy-metal stress. In parallel, the Cluster of Orthologous Groups-level relative abundances of heavy-metal efflux-related functions, including the ATP-dependent ZntA-family P-type ATPase as well as other cation and arsenite efflux transporters, were higher before remediation and declined thereafter (Table S4). Oxidative stress defense genes, including *trxR*, *cat*, and *katG*, followed the same overall trend (Table S5). Consistently, the relative abundances of genes encoding ATP-dependent ZntA-family P-type ATPases and several oxidative stress defense genes were significantly and positively correlated with BA-HM concentrations and soil respiration rates across the remediation process (Figs S8–S9). These results suggest that heavy-metal stress is associated with greater microbial functional potential for energy production, metal detoxification, and oxidative stress defense. Because the TCA cycle supplies reducing equivalents to oxidative phosphorylation for ATP production, the concurrent increases in genomic potential for respiratory energy metabolism and ATP-dependent metal efflux, together with higher measured respiration, are consistent with the hypothesis that heavy-metal detoxification imposes additional energetic demands that may contribute to elevated CO₂ release. As heavy-metal stress was progressively alleviated during remediation, declines in the relative abundances of genes associated with stress response and metal detoxification, together with the measured increases in microbial growth and CUE, indicated that microbial communities shifted from energy-intensive physiological detoxification towards growth. Together, these results suggest that the remediation-driven decrease in CO₂ emissions, coupled with sustained increases in microbial CUE, may enhance the potential for soil carbon storage.

### Remediation altered the composition of active bacterial communities

Although the decrease in the functional potential for central carbon metabolism and heavy-metal detoxification during remediation provides a key explanation for the remediation-induced reduction in soil CO₂ emissions, the drastic changes in active microbial community composition may also have contributed to shifts in carbon-cycling processes.

Remediation significantly altered the taxonomic structure of active bacterial communities (Fig. 3A). In non-treated soils, the active microbiota was predominantly composed of *Paenibacillus*. During the mid-stage of remediation, *Arthrobacter*, *Microvirga*, and *Bacillus* became dominant. By the final stage, *Microvirga* abundance increased further, with *Arthrobacter*, *Microvirga*, and *Methylobacterium-Methylorubrum* identified as the main taxa (Fig. 3B). These successional patterns reflect a shift in the life-history strategies of active bacterial communities during soil remediation. We used average rRNA operon (*rrn*) copy number as a community-level proxy for bacterial life-history strategy, given its association with maximal growth rate in response to resource availability [27]. Community-average *rrn* copy number declined significantly over the course of remediation (Fig. 3C), indicating a shift in the active bacterial community from high-*rrn*, disturbance-responsive opportunistic taxa toward low-*rrn* taxa with more conservative and resource-efficient life-history strategies. In heavy metal-contaminated soils, taxa with higher growth potential may be selectively favored because their capacity for rapid resource acquisition and metabolic response can help sustain energetically costly metal-detoxification and stress-defense processes. However, this higher genomic growth potential does not necessarily translate into higher realized growth rates under metal stress (Fig. 2D), because a larger fraction of assimilated resources is likely diverted from biomass production toward detoxification, repair, and maintenance. As remediation alleviated metal toxicity and reduced the metabolic burden of stress responses, low-*rrn* taxa may have gained a competitive advantage by adopting more efficient resource-use strategies. Community-average *rrn* copy number was significantly negatively correlated with microbial CUE (Fig. S10), linking this life-history strategy transition to the enhanced potential for soil carbon storage in remediated soil. Microbial community stability describes the ability of microbial community composition to withstand environmental perturbations and recover toward its pre-disturbance state, encompassing both resistance and resilience [28]. The ratio of negative to positive cohesion, which is commonly used to characterize microbial community stability [26], increased significantly during remediation, suggesting that the active bacterial community developed a more stable and resilient organization. This enhanced community stability may facilitate the rapid recovery of the active bacterial community from heavy-metal stress (Fig. 3D).

**Figure 3:**
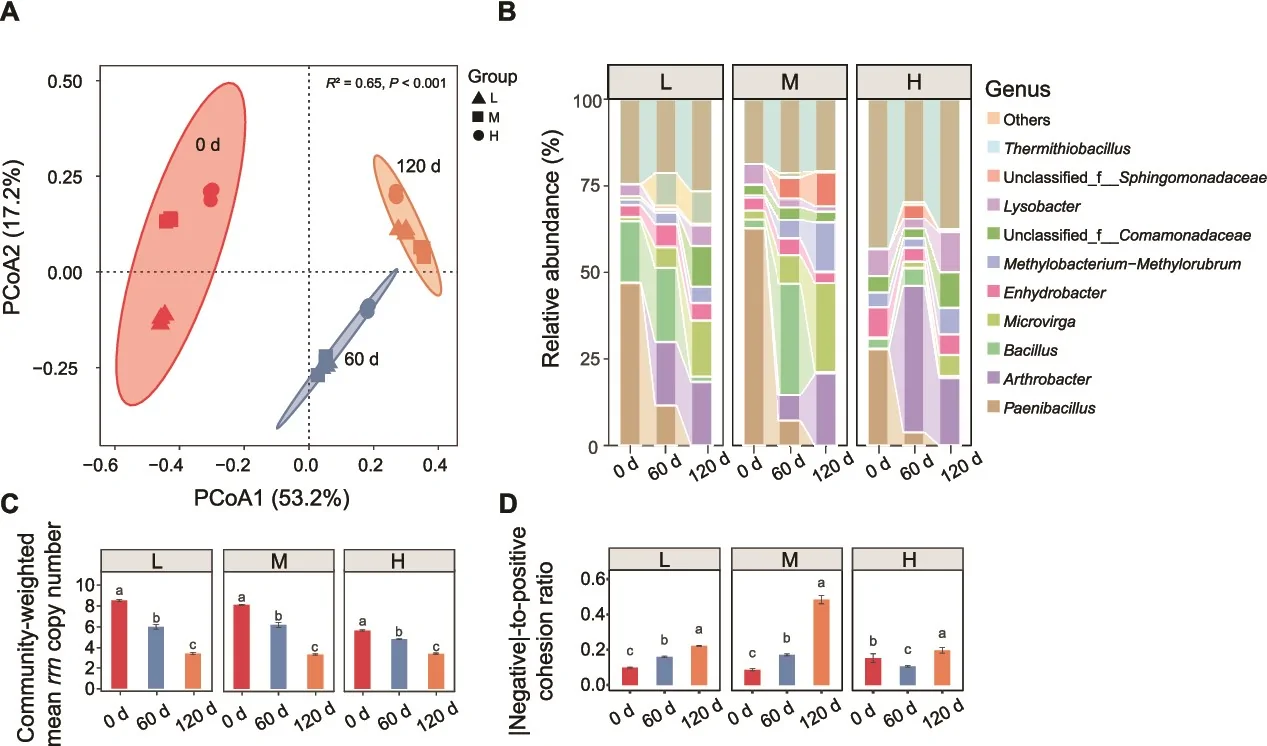
Effects of remediation on active bacterial community traits. Active bacterial communities were characterized by 16S rRNA gene amplicon sequencing of FDAA-positive bacterial cells isolated by fluorescence-activated cell sorting (FACS). (A) PCoA of community composition based on Bray–Curtis dissimilarity (PERMANOVA: *R*^2^ = 0.65, *P* < .001); ellipses indicate 95% confidence intervals grouped by sampling time (red, Day 0; blue, Day 60; orange, Day 120). Shapes represent contamination levels (triangle, L; square, M; circle, H). (B) Dominant active bacterial genera in slightly (L), moderately (M), and heavily (H) contaminated soils across the remediation period. Taxa are ordered by their relative abundance across remediation treatments. (C) Community-weighted mean *rrn* copy number, reflecting the life-history strategy of the active bacterial community, in slightly (L), moderately (M), and heavily (H) contaminated soils across the remediation period. (D) |Negative|-to-positive cohesion ratio, reflecting the stability of the active bacterial community, in slightly (L), moderately (M), and heavily (H) contaminated soils across the remediation period. Bars represent the mean ± SD (*n* = 3). Different letters above the bars indicate significant differences among treatments, as determined by one-way ANOVA followed by Tukey’s HSD test (*P* < .05).

### Single-cell Raman spectroscopy reveals physiological dynamics associated with microbial carbon use efficiency

We next investigated how shifts in physiological traits regulated microbial CUE, given its pivotal role in controlling SOC accumulation following remediation. We used D₂O probing coupled with single-cell Raman spectroscopy to directly profile microbial physiological traits during remediation, including the active bacterial fraction, single-cell metabolic activity, and intracellular biomolecular composition.

Throughout the 120-day remediation period, the proportion of active bacteria increased significantly across all three contamination levels. Moreover, the proportion of metabolically active cells detected by Raman-D₂O ranged from 69.3% to 98.9% (Fig. 4A, Fig. S11). These results suggest that Raman-D₂O captures a broad physiologically active fraction of the soil bacterial community, allowing a comprehensive assessment of the physiological state of active bacteria.

**Figure 4:**
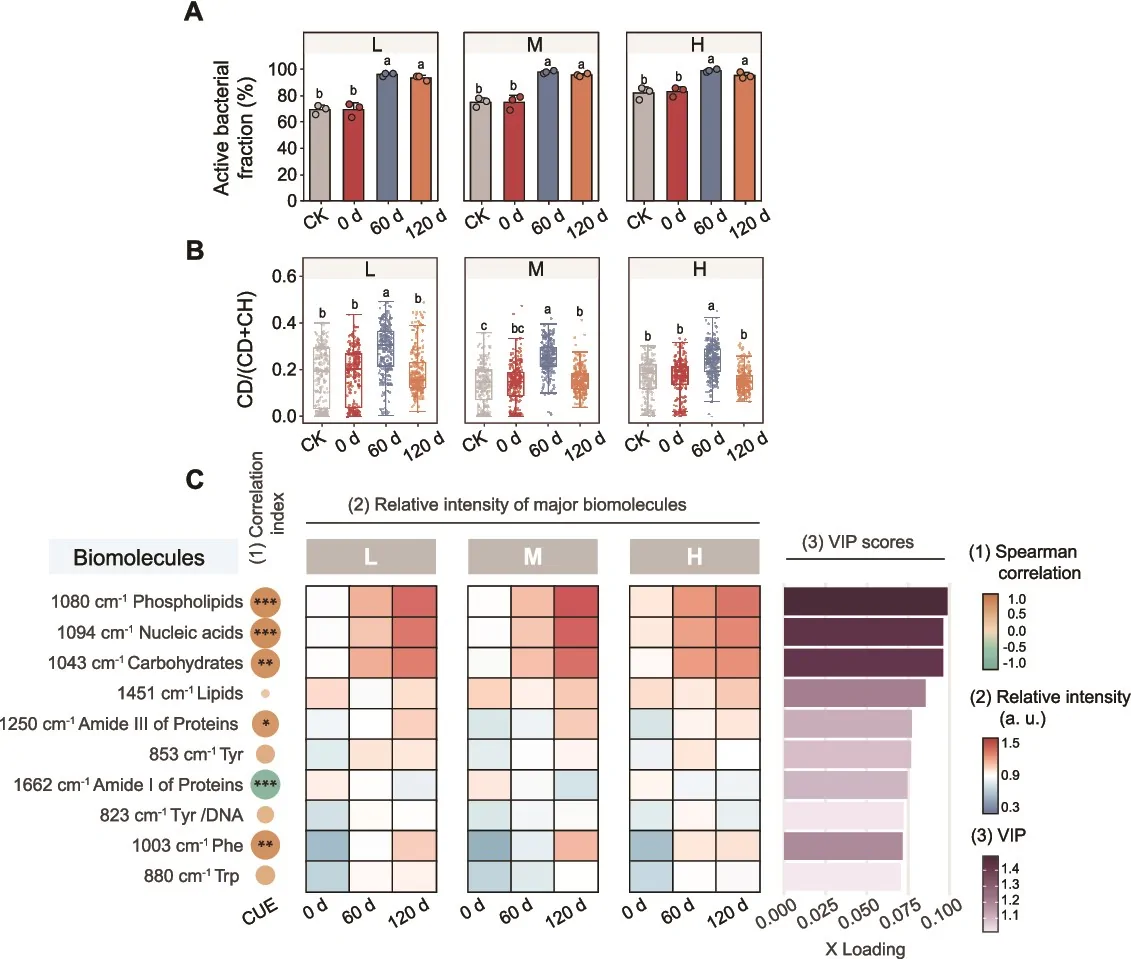
Physiological dynamics of bacteria and Raman spectral markers predicting CUE. (A) Proportion of metabolically active bacteria detected by single-cell Raman-D₂O. Bars represent the mean ± SD (*n* = 3). Different letters above the bars indicate significant differences among treatments, as determined by one-way ANOVA followed by Tukey’s HSD test (*P* < .05). (B) Temporal dynamics of bacterial metabolic activity during remediation. Each dot in the box plots represents an individual bacterial cell. Different letters above the box indicate significant differences among treatments, as determined by one-way ANOVA followed by Tukey’s HSD test (*P* < .05). (C) Key Raman spectral markers predicting microbial CUE. From left to right: bubble plot showing Spearman correlations between Raman-assigned biomolecular features and CUE, bubble size represents the absolute Spearman correlation coefficient; heatmap displaying the relative intensities of major biomolecules contributing to CUE variation; bar plots of biomolecular loadings on the first (most explanatory) PLSR latent variable for CUE. Bar color encodes variable-importance-in-projection (VIP) scores. Significance: ^*^P < .05, ^**^P < .01, ^***^P < .001. Correlations without asterisks are not statistically significant (*P* ≥ .05). CK represents the contaminated control soil without remediation material amendment, which was planted with ryegrass from Day 60 to Day 120 and sampled at Day 120. Tyr, tyrosine; Phe, phenylalanine; Trp, tryptophan.

We also tracked changes in the metabolic activity of indigenous microbes throughout the remediation process. Across all three soils spanning different contamination levels, microbial metabolic activity followed a consistent non-linear trajectory, increasing from Day 0 to Day 60 and then declining from Day 60 to Day 120 (Fig. 4B). The distribution of microbes across different metabolic activity states progressively shifted towards intermediate-to-low activity states during remediation (Fig. S12). This trend raises the possibility that the increasing abundance of relatively low-activity bacteria may be associated with a more efficient mode of carbon cycling.

Microbial CUE is closely linked to how exogenous carbon, once assimilated by microorganisms, is processed through intracellular catabolism, anabolism, and reallocation. Given the high complexity and diversity of bacterial intracellular biomolecules, Raman fingerprinting offers a semi-quantitative approach for characterizing key cellular biomolecular components. To identify which intracellular molecular components are most strongly associated with changes in CUE, we applied PLSR to link Raman spectral features with measured CUE values. Our analysis identified phospholipids (1080 cm^−1^), nucleic acids (823, 1094 cm^−1^), carbohydrates (1043 cm^−1^), amino acids (823, 853, 880, 1003 cm^−1^), protein (1250, 1662 cm^−1^), and lipid (1451 cm^−1^) as major contributors to CUE variation. Phospholipids, nucleic acids, and carbohydrates showed the strongest contributions to CUE shifts, and their abundances increased progressively during remediation. This pattern suggests a microbial shift towards enhanced anabolic investment and biomass construction. Further correlation analysis revealed that phospholipids (1080 cm^−1^), nucleic acids (1094 cm^−1^), carbohydrates (1043 cm^−1^), amino acids (1003 cm^−1^), and protein (1250 cm^−1^) were significantly positively correlated with CUE, whereas protein (1662 cm^−1^) showed significant negative correlations (Fig. 4C). The divergent responses of these two protein-associated bands primarily reflect shifts in intracellular protein composition during remediation, with the 1250 cm^−1^ feature assigned to the amide III region and the 1662 cm^−1^ feature corresponding to the amide I region. Genes encoding proteins involved in oxidative stress defense and heavy metal efflux decreased progressively during remediation. Together, these patterns indicate that bacterial protein synthesis may have shifted from stress-resistance-related proteins towards growth-related proteins during remediation.

### Active bacterial physiological traits are the strongest direct predictors of microbial carbon use efficiency

The soil carbon storage potential is primarily determined by microbial CUE, whereas microbial CUE is an emergent and dynamic property jointly shaped by multiple underlying traits [14, 29]. Given the complexity and diversity of microbial traits, we partitioned them into community-level traits (i.e. active bacterial community life history and community stability) and physiological traits (i.e. intracellular biomolecular content, metabolic activity, and proportion of active bacteria), and used structural equation modelling (SEM) to disentangle their relative contributions to variation in CUE (Fig. 5, Table S6). SEM results revealed that microbial physiological traits were the strongest direct predictors of microbial CUE. Specifically, microbial CUE was negatively affected by microbial metabolic activity (standardized effect size, SES = −0.4181, *P* = .0041) but positively affected by the proportion of active bacteria (SES = 0.3872, *P* = .0529) and intracellular biomolecular content (SES = 0.7359, *P* = .0002). In contrast, microbial community traits showed comparatively weak direct effects on microbial CUE, as indicated by the negligible effects of active bacterial life-history strategy (SES = 0.0008, *P* = .9966) and community stability (SES = −0.1075, *P* = .2861). However, the life-history strategy of active bacteria could indirectly influence microbial CUE by affecting the proportion of active bacteria (SES = −0.3491, *P* = .0244), bacterial metabolic activity (SES = 0.7283, *P* = .0159), and intracellular biomolecular content (SES = −0.6314, *P* < .0001). This likely reflects the fact that microbial life-history strategies represent coordinated trade-offs among growth, resource acquisition, and stress tolerance, which regulate the allocation of assimilated C to growth-related anabolism and thereby shape microbial CUE. Moreover, compared with soil resource availability (DOC, NH_4_^+^, and AP) and pH, BA-HMs showed the strongest direct effects on microbial physiological traits. This suggests that shifts in BA-HMs during soil remediation play a key role in reshaping microbial physiological traits, thereby changing microbial CUE.

**Figure 5:**
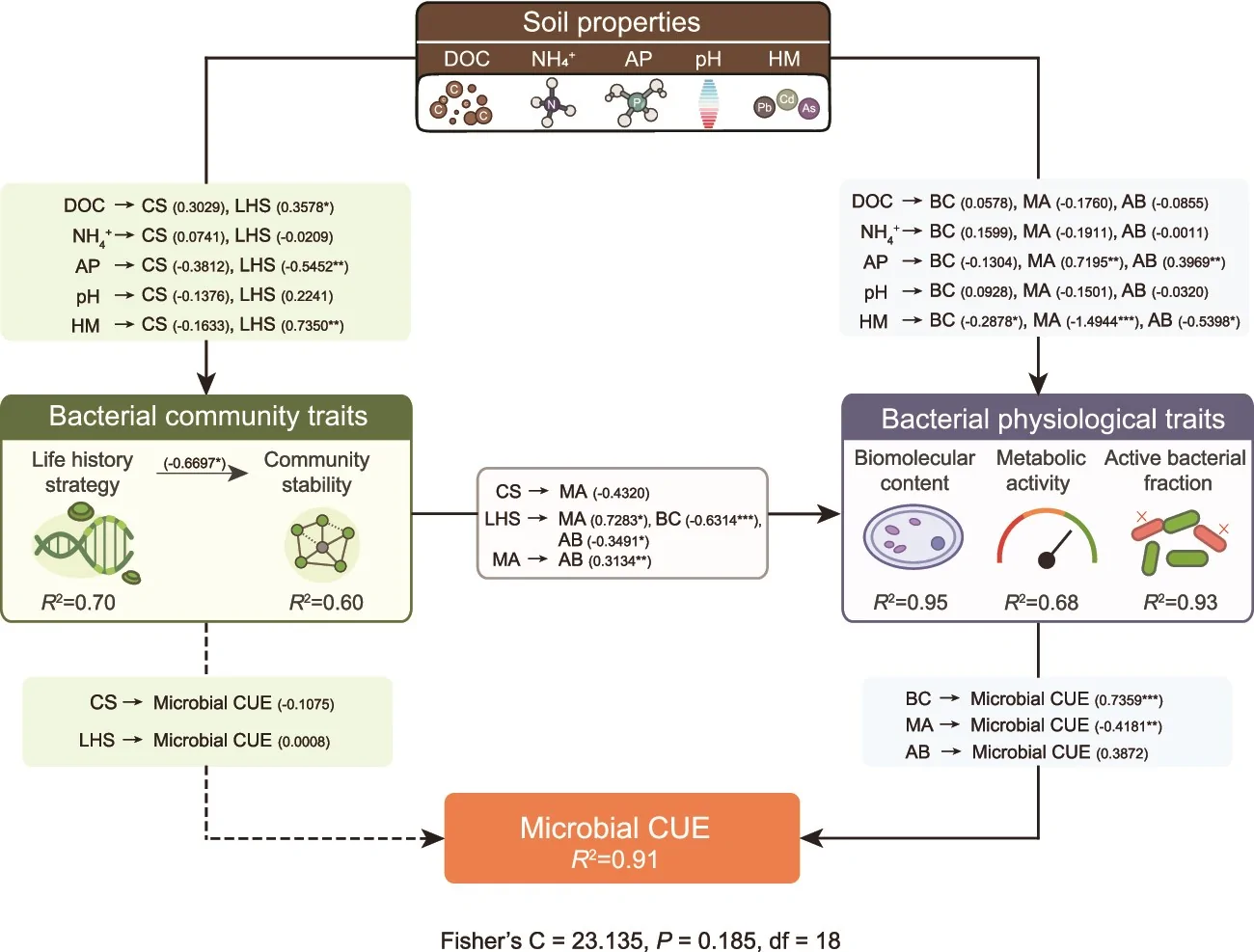
Structural equation model illustrating the relative contributions of soil properties, bacterial community traits, and bacterial physiological traits to CUE. Standardized path coefficients are shown in parentheses. Asterisks denote significance levels: ^*^P < .05, ^**^P < .01, ^***^P < .001. The proportion of explained variance (*R*^2^) is shown for each response variable. Dashed paths indicate nonsignificant relationships (*P* > .05). Soil properties include DOC, NH₄^+^, available P (AP), pH, and heavy metals (HM: PC1 from a PCA of bioavailable Pb, Cd, and As). Bacterial community traits include community stability (CS; ratio of |negative|:positive cohesion) and life history strategy (LHS; community-weighted mean *rrn* copy number of the active bacterial community). Bacterial physiological traits include the biomolecule content (BC; PC1 of key Raman features predicting CUE identified by PLSR), metabolic activity (MA), and active bacterial fraction (AB). Overall model fit: Fisher’s C = 23.135, *P* = .185, df = 18.

## Discussion

Heavy metal contamination affects soil ecosystems in ways that extend well beyond pollutant accumulation, with profound consequences for soil carbon cycling. Our results show that long-term heavy metal contamination was associated with substantially greater microbial respiratory C loss, whereas short-term remediation reduced this loss and increased microbial CUE, indicating enhanced soil carbon storage potential. By resolving microbial functional potential, active bacterial succession, and physiological traits across the remediation trajectory, our study reveals that microbial adaptation to heavy metal stress contributes to elevated soil CO₂ emissions in contaminated soils, whereas the physiological regulation of active bacteria during remediation underpins the recovery of soil carbon storage potential. These findings underscore the necessity of incorporating microbial physiological traits into the monitoring framework of heavy metal-contaminated site remediation, improving assessments of soil functional recovery and supporting the long-term sustainability of soil resources.

### Heavy metal contamination accelerates microbial respiratory carbon loss from soil

Due to the well-known toxicity of heavy metals, previous studies have generally concluded that heavy metal contamination inhibits microbial respiration in soils [30, 31]. However, our findings challenge this assumption: Compared with adjacent uncontaminated soils, heavy metal-contaminated soils exhibited, on average, 79.4% higher cumulative CO₂ emissions (Fig. 1A). In addition, CO₂ emissions from non-treated soils exceeded those from remediated soils by 58.0%, and were highest in heavily contaminated soils. These results suggest that heavy metal contamination accelerates microbial respiratory carbon loss from soil.

A key reason for the discrepancy between our findings and previous studies lies in the different temporal scales considered. Most prior research has focused on the short-term effects of heavy metal contamination, typically spanning only weeks to months [31, 32], during which acute metal toxicity primarily suppresses metabolic activity or causes mortality among susceptible soil microorganisms. In contrast, microbes possess the capacity to evolve in response to environmental pressures over longer timescales [33]. The soils examined in our study have experienced decades of heavy metal exposure, providing ample opportunity for selective pressure to drive the evolution of resistant populations. As a result, a subset of microbes has acquired metal resistance traits and continues to thrive under chronic contamination. This is supported by our observation that 69.3% to 83.0% of the bacterial populations in polluted soils remained metabolically active (Fig. 4A).

Microbial metabolic processes associated with heavy metal resistance are highly energy-intensive. Upon entry into microbial cells, heavy metals induce oxidative stress, and microbial resistance commonly involves ATP-dependent heavy metal efflux systems and antioxidant defenses, including the thioredoxin system and catalase, both of which require substantial cellular resources and energy investment. Consistent with this interpretation, contaminated soils harbored significantly higher relative abundances of genes encoding antioxidant enzymes, including TrxR, CAT, and KatG, as well as heavy metal efflux pumps such as ZntA-family P-type ATPases, than remediated soils, indicating greater functional potential for oxidative stress defense and metal detoxification. Oxidative phosphorylation serves as the primary pathway for microbes to generate ATP and is typically coupled to the TCA cycle, a central respiratory pathway contributing to bacterial CO₂ release. Taken together, the substantial energetic costs of heavy metal resistance and the significant positive correlations between the relative abundances of metal-detoxification genes and microbial respiration rates, collectively, suggest that greater energetic investment in metal resistance may contribute to enhanced respiratory carbon loss under chronic contamination. Another plausible mechanism is that Cd, owing to its strong thiophilicity, may disrupt Fe-S proteins and Fe-S cluster homeostasis [34]. Fe-S clusters play a critical role in respiratory electron transfer [35]. Such disruption may promote electron leakage and reduce electron transport efficiency. This may in turn increase the demand for reducing equivalents generated through the TCA cycle to support oxygen reduction and energy production, thereby contributing to higher carbon loss from contaminated soils. Given that soil ecosystems are increasingly exposed to multiple global change stressors, including drought and heatwaves [36], these stressors may further amplify soil carbon loss when acting synergistically with heavy metal contamination.

### Remediation enhances soil carbon storage potential through reduced respiratory carbon loss and greater microbial carbon retention

Soils constitute the largest terrestrial reservoir of organic carbon, and changes in the balance between carbon inputs and losses can therefore have important consequences for terrestrial carbon storage [37–39]. Although heavy metal contamination accelerated microbial respiratory carbon loss from soil, remediation reduced soil CO_2_ emissions while increasing microbial CUE (Fig. 2B, Fig. 2E). These results suggest that remediation may enhance soil carbon storage potential. Given the widespread occurrence of heavy metal-contaminated sites worldwide, deploying region-specific remediation technologies may be critical for enhancing soil carbon storage potential and reversing trajectories of soil degradation.

Microbial metabolism plays a central role in regulating the fate of organic carbon in soils [40, 41]. It governs both organic matter decomposition and the transformation of assimilated carbon into microbial biomass and metabolites that can contribute to SOC formation and stabilization [42]. Microbial heterotrophic respiration is a major catabolic process through which microorganisms generate energy to support growth, cellular maintenance, and other metabolic functions, while simultaneously releasing organic carbon from soil as CO₂. Our study revealed that chemical remediation significantly reduced cumulative soil CO₂ emissions by 24.2%–51.2%, accompanied by consistent increases in microbial growth rates and MBC. This pattern may primarily reflect the remediation-induced decline in bioavailable metal concentrations, which likely alleviated microbial metal stress and lowered the energetic costs associated with metal detoxification and stress-related cellular maintenance. Together, these responses suggest that alleviation of metal stress was accompanied by a shift in microbial carbon allocation toward growth rather than respiratory loss.

Changes in SOC stocks are generally recognized as the most direct indicators of soil carbon accumulation status. However, SOC accumulation is inherently slow, governed by the balance between plant-derived carbon inputs and microbial decomposition, and typically requires years to become detectable. Given the practical necessity of rapid redevelopment following remediation of heavy-metal contaminated sites, most remediation activities span only a few months, limiting the effectiveness of SOC measurement as an immediate indicator of carbon storage potential. In this context, variations in microbial CUE during remediation may provide a more sensitive indicator of changes in soil carbon storage potential over relatively short timescales. Previous studies have shown that microbial CUE exerts at least four times greater influence than other factors, such as carbon input partitioning, abiotic carbon transfer, substrate decomposition, environmental modifications, and vertical transport, in determining SOC dynamics [7]. In our study, microbial CUE increased progressively during the remediation process, further supporting the notion that successful remediation can enhance soil carbon storage potential by increasing microbial retention of assimilated carbon. Soil is a dynamic system that continues to evolve over time, and the effects of remediation on soil carbon storage potential may extend beyond immediate changes in carbon fluxes and influence longer-term trajectories of carbon accumulation. Such sustained carbon accumulation may, in turn, provide a foundation for the progressive recovery of soil structure and function during subsequent soil development. Considering the central role of SOC in sustaining soil functions [5], remediation efforts that enhance SOC storage potential are essential for strengthening soil resilience and promoting soil development, ultimately preserving the safe and habitable operating space for humanity.

### Active bacterial physiological traits drive enhanced soil carbon storage potential

Trait-based frameworks are increasingly applied in microbial ecology to link microbial traits with ecosystem functions [43–45]. Advances in multi-omics and stable isotope probing technologies have further highlighted that the composition and metabolism of microbial communities are key microbial traits shaping soil carbon storage [46, 47]. However, most current approaches characterize microbial traits at the community level or assess functional potential using metagenomic data, leaving physiological variation among individual active cells largely unresolved. Microbial populations exhibit substantial cell-resolved physiological heterogeneity, including variation in metabolic activity and intracellular biochemical composition [48]. However, community-averaged measurements obscure physiological variation among individual cells, making it difficult to determine how such cell-to-cell heterogeneity contributes to community-level ecological processes. Therefore, bulk measurements of microbial CUE integrate carbon allocation across the community and cannot determine whether a community-level response reflects broadly similar physiological shifts among active cells or is driven by a subset of highly active cells. Such cell-to-cell physiological heterogeneity may therefore represent an underappreciated dimension of microbial carbon allocation and may influence how community-level carbon metabolism responds to environmental perturbation. Recent cell-resolved physiological approaches now make it possible to identify, phenotypically characterize, and selectively recover active individual cells from complex microbial communities. Fluorescent D-amino-acid labeling coupled with flow cytometric sorting identifies and recovers active cells, enabling community traits to be characterized specifically within the active fraction. In parallel, Raman-D₂O probing quantifies the incorporation of deuterium into newly synthesized cellular biomass and reveals cell-to-cell variation in metabolic activity and biomolecular composition. Integrating these cell-resolved measurements with bulk measurements of microbial CUE enables a more realistic and fine-grained characterization of microbial response patterns underlying soil carbon-cycle processes, potentially reducing uncertainty in predictions of microbially mediated carbon-cycle processes. More broadly, this framework could be applied to evaluate how environmental change and diverse pollutants redistribute metabolic activity and biomolecular investment among microbial cells, thereby altering community-level carbon retention.

Given the complexity and multidimensional nature of microbial traits, we classified them into two broad categories: physiological traits (i.e. intracellular biomolecular content, metabolic activity, and proportion of active bacteria) and community-level traits (i.e. active bacterial community life history and community stability). Our findings reveal that physiological rather than community-level traits are the key drivers of CUE variation. This pattern may reflect functional redundancy and phenotypic plasticity, which can partially decouple community-level characteristics from realized carbon-cycling processes. Based on single-cell Raman spectral fingerprints, we identified the major biomolecules contributing to the increase in microbial CUE, including membrane phospholipids, nucleic acids, and carbohydrates. Increases in intracellular membrane phospholipid, nucleic acid, and carbohydrate content directly reflect enhanced biosynthetic processes, such as membrane formation, nucleic acid replication, and polysaccharide storage, which promote the preferential allocation of carbon resources to biomass rather than respiratory losses, thereby enhancing microbial CUE. In addition, the progressive increase in the intracellular protein-associated Raman band at 1250 cm^−1^ also contributed to the increase in microbial CUE during remediation. Given the high affinity of proteins for reactive soil mineral surfaces, partly through coordination between surface-exposed nitrogen-containing groups and mineral-bound metal centers [49, 50], the progressive enrichment of intracellular proteins may increase the potential of microbial biomass to be stabilized as mineral-associated organic carbon during microbial necromass formation, thereby enhancing the persistence of newly sequestered carbon. Moreover, although bacterial metabolic activity was significantly negatively associated with microbial CUE, the average proportion of active bacterial cells increased from 75.7% to 94.7% during remediation, suggesting a shift in microbial carbon cycling from being supported by a smaller active fraction of the bacterial community under contamination to involving a larger fraction of the community after remediation. Such a shift may represent an important feature of soil ecological recovery, because the broader participation of active bacterial cells could reduce reliance on a comparatively smaller subset of the community, thereby potentially enhancing the robustness and resilience of microbial carbon cycling to future environmental perturbations and ultimately contributing to the sustained enhancement and long-term stability of soil carbon storage during soil ecosystem recovery.

## Conclusion

Our study demonstrates that the stress tolerance of soil microorganisms under chronic heavy metal stress disrupts the natural trajectory of soil carbon cycling, thereby accelerating microbial respiratory carbon loss. By simultaneously reducing BA-HMs, suppressing CO₂ emissions, increasing microbial CUE, and promoting the sustained accumulation of MBC, soil remediation enhanced the potential for soil carbon storage. Given that industrial contamination now affects millions of sites worldwide and continues to expand across the Anthropocene, integrating soil carbon fluxes and microbial physiological traits into remediation monitoring will be critical for evaluating functional recovery potential and guiding effective strategies that sustain the long-term development of soil ecosystems.

## Supplementary Material

Supplemental_Material_wrag250

## Data Availability

The metagenomic sequencing data generated in this study have been deposited in the NCBI Sequence Read Archive (SRA) under BioProject accession PRJNA1332155. The raw 16S rRNA gene sequencing data are available in the NCBI SRA under BioProject accession PRJNA1474887. Both datasets are publicly available. Additional data supporting the findings of this study are provided in the Supporting Information.
