# Supplementary material for: Microbial physiological trait shifts link heavy metal remediation to enhanced soil carbon storage potential
Source: ISME J. 2026 Sep 21;20(1):wrag250. doi: 10.1093/ismejo/wrag250 (PMC13657045; doi:10.1093/ismejo/wrag250)
Supplement: Supplementary file 1 [file supplemental_material_wrag250.docx]

**Supporting Information**

# Microbial physiological trait shifts link heavy metal remediation to enhanced soil carbon storage potential

Rui Xue^1, 2^, Jian Li^3^, Shilin Hu^1, 4^, Jiazhi Ding^1, 2^, Cheng Wang^5^, Wenshun Ke^6^, Chaoran Li^7^, Philippe Corvini^8^, Li Cui ^1, *^

*^1^State Key Laboratory of Regional and Urban Ecology, Institute of Urban Environment, Chinese Academy of Sciences, Xiamen 361021, Fujian, China;*

*^2^University of Chinese Academy of Sciences, 19A Yuquan Road, Beijing 100049, China;*

*^3^Ministry of Education Key Laboratory of Ecology and Resource Use of the Mongolian Plateau & Inner Mongolia Key Laboratory of Grassland Ecology, School of Ecology and Environment, Inner Mongolia University, Hohhot 010021, China;*

*^4^Department of Environmental Science and Engineering, University of Science and Technology of China, Hefei 230026, China;*

*^5^Environmental Microbiomics Research Center, School of Environmental Science and Engineering, Southern Marine Science and Engineering Guangdong Laboratory (Zhuhai), Sun Yat-sen University, Guangzhou, 510006, China;*

*^6^College of Environment and Ecology, Hunan Agricultural University, Changsha 410128, China;*

*^7^State Key Laboratory of Crop Stress Adaptation and Improvement, Henan University, Kaifeng 475004, China;*

*^8^Institute for Chemistry and Bioanalytics, FHNW University of Applied Sciences and Arts Northwestern Switzerland, Muttenz, Switzerland*

*Corresponding author. Li Cui, State Key Laboratory of Regional and Urban Ecology, Institute of Urban Environment, Chinese Academy of Sciences, 1799 Jimei road, Xiamen, Fujian 361021, China. E-mail addresses: [lcui@iue.ac.cn](mailto:lcui@iue.ac.cn).

**This file includes:**

Supplementary Methods 1 to 2

Supplementary Figures 1 to 12

Supplementary Tables 1 to 6

**Supplementary Methods**

**Text S1. Measurement and calculation of soil microbial carbon use efficiency (CUE)**

At harvest, 12 mL of headspace gas was withdrawn using a syringe and injected into pre-evacuated 12 mL exetainers. Soil from each vial was then divided into two subsamples: one was fumigated with ethanol-free chloroform for 24 h and subsequently extracted with 0.05 M K₂SO₄; the other was extracted immediately without fumigation. All K₂SO₄ extracts were split into two aliquots: one for DOC analysis and the other for ^13^C quantification, alongside headspace gas samples.

CUE was calculated as follows:

$$atom\% MBC=\frac{[\left( atom\% F DOC\times F DOC \right)-\left( atom\% NF DOC\times NF DOC \right)]}{(F DOC-NF DOC)} (1)$$

$$\%{}^{13}{MBC}=\frac{(atom\%{MBC}_{t}-atom\% {MBC}_{c})}{\left( atom\% sol-atom\%{MBC}_{c} \right)}\times100 \left( 2 \right)$$

$${}^{13}{MBC}=\left( F DOC-NF DOC \right)\times\% {}^{13}{MBC}\div100\div0.45 \left( 3 \right)$$

$$Growth rate=\frac{{}^{13}{MBC}}{t} \left( 4 \right)$$

$${}^{13}{C-{CO}_{2}}=\frac{C_{V}\times V\times273}{V_{m}\times DW\times298\times1000}\times13 \left( 5 \right)$$

$$CUE=\frac{{}^{13}{MBC}}{{}^{13}{MBC}+{}^{13}{C-{CO}_{2}}} \left( 6 \right)$$

In these equations, atom% F DOC and F DOC represent the ^13^C atom percentage and C concentration (μg C g⁻¹ soil) of the fumigated K₂SO₄ extracts, respectively, whereas atom% NF-DOC and NF-DOC denote the corresponding values for the non-fumigated extracts. Atom% MBC_t_ and atom% MBC_c_ refer to the ^13^C enrichment of the treatment and the natural-abundance control, respectively. C_V_ is the ^13^CO_2_ concentration derived from the added ^13^C-enriched glucose (ppm), V is the headspace volume of the incubation vial (mL), DW is the soil dry weight (g), and t is the incubation time (d). V_m_ denotes the molar volume of an ideal gas (L mol^−1^) under standard conditions (273 K, 1 atm). The term 298 K accounts for the incubation temperature, and the factor 1000 was included for unit conversion. Atom% sol represents the isotopic abundance of the glucose substrate (25 atom%). ^13^C-CO₂ refers to the cumulative amount of glucose-derived ^13^C-labeled CO_2_ released during incubation (μg ^13^C-CO_2_ g^−1^ soil) (1).

**Text S2. Metagenomic assembly and functional annotation**

After quality filtering and trimming, high-quality reads from all samples were pooled and co-assembled *de novo* using MEGAHIT v1.2.9 (2). Contigs longer than 500 bp were retained for open reading frame (ORF) prediction using Prodigal v2.6.3 (3), and the predicted coding sequences were translated into amino acid sequences. Predicted ORFs were clustered using CD-HIT v4.8.1 at 95% sequence identity and 90% alignment coverage (4), with the longest sequence in each cluster retained as the representative sequence to construct a nonredundant (NR) gene catalog. To quantify gene abundance, clean reads from each sample were mapped back to the NR gene catalog using Bowtie2, with a minimum sequence identity of 95%. Gene abundance was calculated from the number of mapped reads and gene length and normalized as TPM values. The representative sequences were functionally annotated against the Kyoto Encyclopedia of Genes and Genomes (KEGG) and eggNOG databases using DIAMOND v0.8.35. Hits with an E-value < 1 × 10⁻⁵ and a bit score > 60 were retained, and the highest-scoring hit for each query sequence was used for functional assignment.

**Supplementary Figures**

 **Fig. S1. Temporal changes in the absolute abundance of active bacteria during the remediation process.** Absolute abundances of active bacterial cells quantified by fluorescent D-amino acid (FDAA) labeling in slightly (L), moderately (M), and heavily (H) contaminated soils at 0, 60, and 120 d of remediation. Bars represent the mean ± SD (n = 3).

**Fig. S2. Fluorescence threshold determination for FACS of FDAA-labeled active bacteria.** Representative flow cytometry histograms showing FDAA-labeled samples (red) and unlabeled controls (blue), used to define the fluorescence gate for sorting active bacterial cells in slightly (L), moderately (M), and heavily (H) contaminated soils during remediation. Percentages indicate the fraction of fluorescence-positive cells within each sample.

**Fig. S3. Temporal changes in bioavailable heavy metals during the remediation process.** Concentrations of bioavailable Pb, Cd, As, and their combined levels (Pb + Cd + As) in slightly (L), moderately (M), and heavily (H) contaminated soils at 0, 60, and 120 d of remediation. ppm values indicate concentrations on a dry-soil mass basis (mg kg⁻¹ dry soil). Bars represent the mean ± SD (n = 3). Different letters indicate significant differences based on one-way ANOVA followed by Tukey’s HSD test (*P* < 0.05).

**Fig. S4. Aboveground dry biomass of ryegrass during the remediation process.** Ryegrass was sown on day 60 and cultivated until day 120 in unremediated and remediated soils under slightly (L), moderately (M), and heavily (H) contaminated conditions. Bars represent the mean ± SD (n = 3). Asterisks indicate significant differences between unremediated and remediated soils within each contamination level based on Student’s t-test (P < 0.001). Biomass is expressed as g kg⁻¹ soil.

**Fig. S5. Effects of nano-FeS amendment on microbial carbon metabolism in three uncontaminated soils.** Three uncontaminated soils (N1-N3) were incubated for 30 days with or without nano-FeS materials. (A) Cumulative CO₂ emissions, (B) microbial respiration rate, (C) microbial growth rate, and (D) microbial carbon use efficiency (CUE). No FeS and FeS denote the unamended control and nano-FeS treatment, respectively. Bars represent the mean ± SD (n = 3). Differences between the No FeS and FeS treatments within each soil were evaluated using two-tailed independent-samples *t*-tests. NS indicates no significant difference between treatments (*P* > 0.05).

**Fig. S6. Responses of microbial biomass and metabolic quotient to remediation.** Soil microbial biomass carbon (MBC) and microbial metabolic quotient (qCO₂) in slightly (L), moderately (M), and heavily (H) contaminated soils under the control (CK) and at 0, 60, and 120 d of remediation. MBC denotes soil microbial biomass carbon, and qCO₂ denotes the microbial metabolic quotient. CK denotes contaminated soil without nano-FeS amendment, sampled on day 120 after ryegrass cultivation from day 60 to day 120. ppm values indicate concentrations on a dry-soil mass basis (mg kg⁻¹ dry soil). Bars represent the mean ± SD (n = 3). Different letters indicate significant differences based on one-way ANOVA followed by Tukey’s HSD test (*P* < 0.05).

**Fig. S7. Spearman correlation analysis between soil microbial respiration rate and decarboxylation-related KOs.** Spearman correlations between respiration rate and the abundances of decarboxylation-related KOs involved in central carbon metabolism. Each point represents an individual soil sample. The black line shows the linear regression fit with 95% confidence intervals. K00031, isocitrate dehydrogenase; K00164, 2-oxoglutarate dehydrogenase E1 component; K00658, 2-oxoglutarate dehydrogenase E2 component; K00161, pyruvate dehydrogenase E1 component subunit alpha; K00162, pyruvate dehydrogenase E1 component subunit beta; K00163, pyruvate dehydrogenase E1 component; K00627, pyruvate dehydrogenase E2 component; K00382, dihydrolipoyl dehydrogenase; and K00033, 6-phosphogluconate dehydrogenase.

**Fig. S8. Spearman correlation analysis between soil microbial respiration rate and oxidative stress defense- and heavy metal efflux-related genes.** Spearman correlations between respiration rate and the abundances of genes involved in oxidative stress defense and heavy metal efflux across all soil samples. Each point represents an individual soil sample. The black line shows the linear regression fit with 95% confidence intervals. K00384, thioredoxin reductase (NADPH); K03671, thioredoxin; K03781, catalase; K03782, catalase-peroxidase; K04564, superoxide dismutase, Fe-Mn family; and COG2217, ZntA-family cation-transporting P-type ATPase.

**Fig. S9. Spearman correlation analysis between bioavailable heavy metals and oxidative stress defense- and heavy metal efflux-related genes.** Spearman correlations between bioavailable heavy metal concentrations (Pb + Cd + As) and the abundances of genes involved in oxidative stress defense and heavy metal efflux across all soil samples. Each point represents an individual soil sample. The black line shows the linear regression fit with 95% confidence intervals. K00384, thioredoxin reductase (NADPH); K03671, thioredoxin; K03781, catalase; K03782, catalase-peroxidase; K04564, superoxide dismutase, Fe–Mn family; and COG2217, ZntA-family cation-transporting P-type ATPase.

**Fig. S10. Relationship between microbial CUE and active bacterial life-history strategy.** Spearman correlation between microbial carbon use efficiency (CUE) and community-weighted mean rRNA operon (rrn) copy number. The solid line indicates the fitted relationship, and the shaded area represents the 95% confidence interval.

**Fig. S11. Threshold determination for identifying metabolically active cells by single-cell Raman analysis.** C-D/(C-D + C-H) ratios of 100 individual cells measured in the no-D₂O (D-free) soil control. The cutoff value (dashed line) used to distinguish active cells from background was defined as the mean + 3 SD of the D-free control distribution.

**Fig. S12. Distribution patterns of cells with different metabolic activity levels during the remediation process.** Kernel density distributions of single-cell Raman-derived metabolic activity, expressed as the C-D/(C-D + C-H) ratio, in slightly (L), moderately (M), and heavily (H) contaminated soils under the control (CK) and at 0, 60, and 120 d of remediation. CK denotes contaminated soil without nano-FeS amendment, sampled on day 120 after ryegrass cultivation from day 60 to day 120. The curves illustrate how cells were distributed among different metabolic activity states within each sample, and the dashed lines indicate the mean activity values.

**Supplementary Tables**

**Table S1. Total concentrations of heavy metals in the soil of L, M, H, N1, N2, and N3.**

| Content | L | M | H | N1 | N2 | N3 |
| --- | --- | --- | --- | --- | --- | --- |
| Cd (mg kg^-1^) | 165.0$\pm12.40c$ | 230.0$\pm7.36$b | 433.6$\pm13.28a$ | $0.29\pm0.02d$ | $0.38\pm0.02d$ | $0.45\pm0.03d$ |
| Pb (mg kg^-1^) | 1949$\pm80.87c$ | 2330$\pm129.60$b | 2856$\pm102.30a$ | $4.1\pm0.12d$ | $8.1\pm0.44d$ | $8.9\pm0.45d$ |
| As (mg kg^-1^) | 500.5$\pm70.24c$ | 798.7$\pm44.82$b | 967.4$\pm41.19a$ | $0.54\pm0.02d$ | $0.66\pm0.03d$ | $0.62\pm0.02d$ |

Values are presented as mean ± SD (n = 3). Different letters indicate significant differences (*P* < 0.05, one-way ANOVA followed by Tukey's HSD test).

**Table S2. TPM-normalized abundances of KEGG pathways related to central carbon metabolism detected in this study.**

| KEGG Pathway | L 0 day | L 60 day | L 120 day | M 0 day | M 60 day | M 120 day | H 0 day | H 60 day | H 120 day |
| --- | --- | --- | --- | --- | --- | --- | --- | --- | --- |
| TCA cycle | 6939.5 ± 110.6a | 5994.8 ± 54.3b | 5273.8 ± 215.6c | 6957.1 ± 81.6a | 6179.8 ± 56.5b | 5456.0 ± 33.2c | 7103.7 ± 59.6a | 6226.2 ± 63.3b | 5789.0 ± 100.5c |
| Fatty acid degradation | 6260.6 ± 75.5a | 4685.4 ± 70.0b | 3856.3 ± 199.0c | 6566.8 ± 55.0a | 4827.5 ± 30.9b | 4444.1 ± 23.7c | 6317.8 ± 46.4a | 4805.6 ± 10.1b | 4284.1 ± 54.1c |
| Glycolysis / Gluconeogenesis | 9130.9 ± 136.5a | 8325.8 ± 108.4b | 6882.7 ± 322.2c | 9170.9 ± 95.1a | 8250.6 ± 101.7b | 7321.1 ± 20.2c | 9311.5 ± 63.3a | 8477.8 ± 67.4b | 7634.2 ± 147.2c |
| Lys degradation | 4665.1 ± 47.5a | 4096.8 ± 54.7b | 3189.9 ± 144.9c | 4855.9 ± 37.1a | 3919.6 ± 15.0b | 3510.8 ± 22.9c | 4721.4 ± 14.4a | 3954.2 ± 4.4b | 3492.6 ± 38.3c |
| Oxidative phosphorylation | 12143.5 ± 119.1a | 12070.3 ± 46.1a | 10918.1 ± 214.0b | 11814.7 ± 92.7a | 11630.2 ± 88.4a | 10810.6 ± 38.3b | 12319.4 ± 59.8a | 12085.7 ± 46.8b | 11439.9 ± 98.8c |
| PP pathway | 4797.8 ± 70.2a | 4845.1 ± 73.0a | 4141.9 ± 156.8b | 4948.1 ± 25.9a | 4587.2 ± 57.1b | 4305.8 ± 24.5c | 4930.9 ± 73.3a | 4794.2 ± 30.1a | 4341.6 ± 81.3b |
| Val, Leu and Ile degradation | 8211.6 ± 88.6a | 6448.5 ± 74.1b | 5404.1 ± 248.8c | 8549.1 ± 66.6a | 6561.1 ± 17.2b | 6043.7 ± 15.7c | 8458.2 ± 75.2a | 6511.4 ± 35.6b | 6002.8 ± 74.5c |

Values are presented as mean ± SD (n = 3). Different lowercase letters indicate significant differences among sampling times (0, 60, and 120 day) within each contamination level (L, M, or H) separately. Statistical comparisons were conducted independently within each contamination level, and *P* values were adjusted using the Benjamini-Hochberg (BH) procedure. Abbreviations: PP, pentose phosphate; Val, valine; Leu, leucine; Ile, isoleucine; Lys, lysine.

**Table S3. TPM-normalized abundances of KEGG orthologs involved in oxidative decarboxylation in central carbon metabolism.**

| KO ID | L 0 day | L 60 day | L 120 day | M 0 day | M 60 day | M 120 day | H 0 day | H 60 day | H 120 day |
| --- | --- | --- | --- | --- | --- | --- | --- | --- | --- |
| K00031 | 212.5 ± 9.0a | 108.1 ± 0.2b | 98.2 ± 8.7b | 210.5 ± 6.4a | 130.3 ± 2.9b | 112.3 ± 4.2c | 215.9 ± 3.2a | 141.9 ± 1.6b | 124.1 ± 3.3c |
| K00164 | 286.4 ± 16.6a | 132.5 ± 5.3b | 96.6 ± 8.1c | 278.5 ± 7.5a | 150.4 ± 10.0b | 128.8 ± 6.6c | 303.8 ± 7.1a | 153.1 ± 3.5b | 121.5 ± 6.2c |
| K00658 | 219.4 ± 7.7a | 136.8 ± 2.1b | 90.4 ± 6.4c | 225.2 ± 2.5a | 139.5 ± 4.4b | 144.5 ± 5.6b | 236.7 ± 4.3a | 159.8 ± 2.3b | 120.7 ± 2.5c |
| K00161 | 256.1 ± 8.9a | 203.8 ± 4.9b | 114.4 ± 6.5c | 266.1 ± 11.2a | 187.5 ± 7.5b | 159.8 ± 5.5c | 273.9 ± 3.2a | 204.6 ± 0.6b | 145.2 ± 3.8c |
| K00162 | 279.6 ± 9.8a | 272.3 ± 5.3a | 155.4 ± 10.1b | 306.3 ± 10.2a | 214.9 ± 6.4b | 177.3 ± 4.1c | 304.4 ± 6.7a | 230.5 ± 3.8b | 165.9 ± 8.4c |
| K00163 | 260.3 ± 8.5a | 136.2 ± 3.2b | 107.5 ± 6.4c | 263.1 ± 3.8a | 141.9 ± 6.6b | 105.1 ± 2.2c | 273.9 ± 4.0a | 157.7 ± 2.0b | 124.4 ± 5.0c |
| K00382 | 473.9 ± 13.1a | 400.5 ± 4.7b | 324.2 ± 18.4c | 484.7 ± 2.9a | 365.6 ± 6.6b | 320.8 ± 5.2c | 485.1 ± 11.0a | 398.7 ± 4.5b | 359.4 ± 12.6c |
| K00627 | 297.1 ± 5.2a | 190.8 ± 5.1b | 175.3 ± 8.4b | 344.5 ± 10.0a | 221.8 ± 6.5b | 166.8 ± 3.3c | 328.1 ± 3.4a | 233.0 ± 3.2b | 195.4 ± 7.3c |
| K00033 | 190.4 ± 6.7a | 102.5 ± 0.7b | 58.3 ± 4.4c | 197.5 ± 5.1a | 98.3 ± 2.4b | 77.5 ± 4.3c | 200.5 ± 6.6a | 118.2 ± 1.5b | 71.5 ± 1.6c |

Values are presented as mean ± SD (n = 3). Different lowercase letters indicate significant differences among sampling times (0, 60, and 120 day) within each contamination level (L, M, or H) separately. Statistical comparisons were conducted independently within each contamination level, and *P* values were adjusted using the Benjamini-Hochberg (BH) procedure. The KEGG KOs shown here represent key enzymatic components of central carbon metabolism. K00031, isocitrate dehydrogenase (IDH; isocitrate to 2-oxoglutarate and CO₂); K00164 and K00658, 2-oxoglutarate dehydrogenase complex components, with K00382 encoding the shared E3 dihydrolipoyl dehydrogenase component involved in NAD⁺-dependent redox recycling; K00161, K00162, K00163 and K00627, pyruvate dehydrogenase complex components, which catalyze pyruvate conversion to acetyl-CoA and CO₂; and K00033, 6-phosphogluconate dehydrogenase (6-PGDH; 6-phosphogluconate to ribulose-5-phosphate and CO₂).

**Table S4. TPM-normalized abundances of selected COGs associated with heavy metal efflux.**

| COG ID | L 0 day | L 60 day | L 120 day | M 0 day | M 60 day | M 120 day | H 0 day | H 60 day | H 120 day |
| --- | --- | --- | --- | --- | --- | --- | --- | --- | --- |
| COG2217 | 8.7 ± 0.5a | 1.7 ± 0.2b | 1.1 ± 0.2b | 11.1 ± 0.6a | 2.3 ± 0.6b | 3.1 ± 0.8b | 14.6 ± 1.4a | 4.0 ± 0.2b | 2.0 ± 0.2b |
| COG1230 | 10.1 ± 1.3a | 3.1 ± 0.6b | 3.4 ± 0.1b | 11.3 ± 0.2a | 3.3 ± 0.5b | 8.2 ± 2.2a | 11.3 ± 1.1a | 5.0 ± 0.9b | 4.0 ± 1.2b |
| COG0053 | 0.2 ± 0.3a | 0.0 ± 0.0a | 0.0 ± 0.0a | 0.1 ± 0.2a | 0.0 ± 0.0a | 0.0 ± 0.0a | 0.4 ± 0.7a | 0.0 ± 0.0a | 0.3 ± 0.3a |
| COG0798 | 10.7 ± 0.7a | 1.9 ± 0.3b | 0.8 ± 0.2b | 22.7 ± 1.4a | 2.4 ± 0.6b | 1.5 ± 0.3b | 14.1 ± 1.3a | 3.2 ± 0.6b | 1.4 ± 0.4b |

Values are presented as mean ± SD (n = 3). Different lowercase letters indicate significant differences among sampling times (0, 60, and 120 day) within each contamination level (L, M, or H) separately. Statistical comparisons were conducted independently within each contamination level, and P values were adjusted using the Benjamini-Hochberg (BH) procedure. COG annotations: COG2217, cation-transporting P-type ATPase (ZntA); COG1230, Co/Zn/Cd efflux system component (CzcD); COG0053, divalent metal cation (Fe/Co/Zn/Cd) efflux pump (FieF); COG0798, Acr3-family arsenite efflux pump.

**Table S5. TPM-normalized abundances of KEGG orthologs involved in antioxidant defense.**

| KO ID | L 0 day | L 60 day | L 120 day | M 0 day | M 60 day | M 120 day | H 0 day | H 60 day | H 120 day |
| --- | --- | --- | --- | --- | --- | --- | --- | --- | --- |
| K00384 | 473.7 ± 10.9b | 495.5 ± 4.8a | 312.1 ± 8.2c | 468.1 ± 10.7a | 386.7 ± 4.1b | 303.7 ± 3.3c | 488.3 ± 6.9a | 401.2 ± 3.6b | 307.5 ± 5.9c |
| K03671 | 425.9 ± 0.3b | 520.1 ± 9.7a | 365.6 ± 29.1c | 458.2 ± 8.3a | 408.4 ± 6.6b | 339.5 ± 3.8c | 445.1 ± 8.5a | 423.3 ± 2.5a | 360.4 ± 12.7b |
| K03781 | 100.6 ± 4.0a | 94.0 ± 1.5a | 58.3 ± 3.1b | 79.8 ± 2.9a | 77.2 ± 2.4a | 70.5 ± 2.2b | 93.5 ± 2.6a | 83.3 ± 1.0b | 57.0 ± 1.9c |
| K03782 | 154.7 ± 7.0a | 83.0 ± 1.7b | 88.3 ± 6.5b | 155.2 ± 2.9a | 102.1 ± 4.3b | 86.9 ± 3.0c | 166.1 ± 3.4a | 108.7 ± 3.5b | 99.2 ± 1.2c |
| K04564 | 127.2 ± 2.8b | 136.9 ± 3.3a | 105.8 ± 3.5c | 124.6 ± 5.9b | 116.5 ± 3.7b | 138.6 ± 4.6a | 135.2 ± 4.7a | 142.4 ± 2.7a | 100.8 ± 1.8b |

Values are presented as mean ± SD (n = 3). Different lowercase letters indicate significant differences among sampling times (0, 60, and 120 day) within each contamination level (L, M, or H) separately. Statistical comparisons were conducted independently within each contamination level, and P values were adjusted using the Benjamini-Hochberg (BH) procedure. The KEGG KOs shown here represent key enzymatic components of oxidative stress responses. Oxidative stress-related KOs include K00384, thioredoxin reductase; K03671, thioredoxin; K03781, catalase; K03782, catalase-peroxidase; and K04564, Fe/Mn-superoxide dismutase.

**Table S6. Standardized path coefficients and indirect effects in the structural equation model.**

| Response | Predictor | Indirect effect | Standardized path coefficients | *P* Value |
| --- | --- | --- | --- | --- |
| Life history strategy | DOC | 0.00000000 | 0.3578 | 0.0320 |
| Life history strategy | NH₄⁺ | 0.00000000 | -0.0209 | 0.9215 |
| Life history strategy | AP | 0.00000000 | -0.5452 | 0.0041 |
| Life history strategy | pH | 0.00000000 | 0.2241 | 0.1441 |
| Life history strategy | HM | 0.00000000 | 0.7350 | 0.0080 |
| Community stability | DOC | -0.23961866 | 0.3029 | 0.1565 |
| Community stability | NH₄⁺ | 0.01399673 | 0.0741 | 0.7675 |
| Community stability | AP | 0.36512044 | -0.3812 | 0.1345 |
| Community stability | pH | -0.15007977 | -0.1376 | 0.4626 |
| Community stability | HM | -0.49222950 | -0.1633 | 0.6471 |
| Community stability | Life history strategy | 0.00000000 | -0.6697 | 0.0171 |
| Metabolic activity | DOC | 0.23324820 | -0.1760 | 0.3896 |
| Metabolic activity | NH₄⁺ | -0.05327926 | -0.1911 | 0.4140 |
| Metabolic activity | AP | -0.39012279 | 0.7195 | 0.0072 |
| Metabolic activity | pH | 0.28748969 | -0.1501 | 0.3935 |
| Metabolic activity | HM | 0.81848924 | -1.4944 | 0.0002 |
| Metabolic activity | Life history strategy | 0.28931040 | 0.7283 | 0.0159 |
| Metabolic activity | Community stability | 0.00000000 | -0.4320 | 0.0500 |
| Biomolecule content | DOC | -0.22591492 | 0.0578 | 0.4556 |
| Biomolecule content | NH₄⁺ | 0.01319626 | 0.1599 | 0.0950 |
| Biomolecule content | AP | 0.34423928 | -0.1304 | 0.1639 |
| Biomolecule content | pH | -0.14149674 | 0.0928 | 0.1863 |
| Biomolecule content | HM | -0.46407900 | -0.2878 | 0.0382 |
| Biomolecule content | Life history strategy | 0.00000000 | -0.6314 | P < 0.0001 |
| Active bacterial fraction | DOC | -0.10696639 | -0.0855 | 0.3540 |
| Active bacterial fraction | NH₄⁺ | -0.06929227 | -0.0011 | 0.9920 |
| Active bacterial fraction | AP | 0.29355614 | 0.3969 | 0.0067 |
| Active bacterial fraction | pH | -0.03517538 | -0.0320 | 0.6816 |
| Active bacterial fraction | HM | -0.46841893 | -0.5398 | 0.0130 |
| Active bacterial fraction | Life history strategy | 0.31891910 | -0.3491 | 0.0244 |
| Active bacterial fraction | Metabolic activity | 0.00000000 | 0.3134 | 0.0032 |
| CUE | Life history strategy | -0.82980346 | 0.0008 | 0.9966 |
| CUE | Community stability | 0.12819666 | -0.1075 | 0.2861 |
| CUE | Metabolic activity | 0.12134848 | -0.4181 | 0.0041 |
| CUE | Biomolecule content | 0.00000000 | 0.7359 | 0.0002 |
| CUE | Active bacterial fraction | 0.00000000 | 0.3872 | 0.0529 |

Life-history strategy was quantified as *rrn* copy number, and community stability as the negative-to-positive cohesion ratio. HM denotes the composite variable represented by the first principal component (PC1) derived from principal component analysis (PCA) of bioavailable Pb, Cd, and As. Biomolecule content denotes the composite variable represented by PC1 derived from PCA of biomolecules associated with variation in CUE.

**References**

1.	Geyer KM, Dijkstra P, Sinsabaugh R et al. Clarifying the interpretation of carbon use efficiency in soil through methods comparison. *Soil Biol Biochem* 2019;128:79-88. https://doi.org/10.1016/j.soilbio.2018.09.036

2.	Li D, Liu CM, Luo R et al. MEGAHIT: an ultra-fast single-node solution for large and complex metagenomics assembly via succinct de Bruijn graph. *Bioinformatics* 2015;31:1674-6. https://doi.org/10.1093/bioinformatics/btv033

3.	Hyatt D, Chen GL, LoCascio PF et al. Prodigal: prokaryotic gene recognition and translation initiation site identification. BMC *Bioinformatics* 2010;11:119. https://doi.org/10.1186/1471-2105-11-119

4.	Li W, Godzik A. Cd-hit: a fast program for clustering and comparing large sets of protein or nucleotide sequences. *Bioinformatics* 2006;22:1658-9. <https://doi.org/10.1093/bioinformatics/btl158>
